# Early Surgery after Coronary Revascularization: A Fine Line Between Bleeding and Thrombosis

**Published:** 2014-12-19

**Authors:** C De Biase, E Capuano, S De Luca, C D’Anna, R Luciano, F Piscione, B Trimarco, G Galasso

**Affiliations:** Department of Advanced Biomedical Sciences, “Federico II” University of Naples, Italy; 1 Department of Medicine and Surgery, University of Salerno, Salerno, Italy e-mail: gengalas@unina.it

**Keywords:** *non-cardiac surgery*, *percutaneous coronary intervention*, *antiplatelet therapy*, *stent thrombosis*, *bleeding*

## Abstract

Management of PCI patients undergoing early surgery is still a matter of debate. Noteworthy, PCI patients require a dual antiplatelet therapy (DAPT), with aspirine and a thienopiridine (clopidogrel, prasugrel, ticagrelor), because of the high risk of stent thrombosis (ST), myocardial infarction (MI) and death, especially within the first month. Indeed, the number of surgical interventions after PCI is actually increasing, and physicians are looking for the best antiplatelet therapy management, in order to reduce both, bleeding and thrombosis risk. In this paper, current guidelines therapy management and new optional strategies to reduce the cardiovascular risk, related to early surgery, are discussed.

## INTRODUCTION

Percutaneous coronary intervention (PCI) is an invasive non-surgical cardiovascular intervention, widely used for the treatment of coronary artery disease (CAD), with high rates of success and low rates of complications (1–3). Since its introduction in the clinical scenario, “new devices” have replaced, or served as adjuncts, to the first plain old balloon angioplasty (POBA), including bare-metal stents (BMS) and drug-eluting stents (DES), with improved efficacy and safety profile of percutaneous revascularization, and consequent transition from the balloon PCI alone to the PCI with stenting (4). However, because of the high thrombotic risk following stent deployment, patients undergoing PCI with stenting require a specific dual antiplatelet therapy (DAPT), with aspirin and a P2Y_12_ receptor antagonist (clopidegrel, prasugrel or ticagrelor), as best medical treatment for preventing thrombosis (5). According to the current PCI guidelines (6–9), in patients with stable CAD, DAPT is strongly recommended for at least 1 month after a BMS deployment or POBA, and up to 6–12 months after a DES, in order to reduce the risk of ischemic events following PCI, including stent thrombosis (ST), myocardial infarction (MI) and death (10–13). Nevertheless, it should be considered that, after acute coronary syndromes (ACS) (ST-elevation-MI, Non-ST-elevation-MI, unstable-angina), the DAPT duration required is at least up to 12 months. Nowadays, there is a prevalence of premature withdrawal of DAPT between 10% and 50% of patients underwent PCI. One of the main reasons of DAPT dysruption is the need of early surgery, defined as surgical procedure occurring within 6 weeks after BMS or within 12 months after DES deployment (14). Interestingly, surgical interventions are required within 2 years in approximately 5% to 15% of patients undergoing PCI (15). Moreover, several studies confirmed that between 4% and 8% of PCI patients require non-cardiac surgery within 1 year from revascularization (16–20). Thereby, the drugs management of patients who need surgery after PCI remains one of the most debated topics. This is relevant not only for interventional cardiologists, but also for general cardiologists, surgeons, anesthesiologists, and primary care physicians, especially because they have to define and to face the life threatening ischemic and bleeding risk (15).

Indeed, surgical interventions are associated with a high bleeding risk in patients on DAPT, thus an early interruption of antiplatelet therapy is often required. Otherwise, the premature discontituation of DAPT exposes patients to the risk of thrombotic consequences, further increased by the pro-inflammatory and pro-thrombotic effects of surgery itself (higher platelets reactivity, coaugulation proteins, fibrinogen levels and anemia or traumathic basal conditions). Current guidelines regarding PCI and surgery (21), suggest interrupting thienopiridine 5 (clopidogrel and ticagrelor) or 7 (prasugrel) days before surgery, without aspirin withdrawal, with exception for intracranial surgery or transurethral prostatectomy. However, the decision to early interrupt, one or both antiplatlet agents, depends on the evaluation of the individual thrombotic/bleeding risk. The aim of this review is to get more inside the therapeutic strategies for CAD patients requiring surgery after PCI with stenting.

## ISCHEMIC COMPLICATIONS IN SURGICAL PATIENTS WITH PREVIOUS PCI

**Figure 1:**
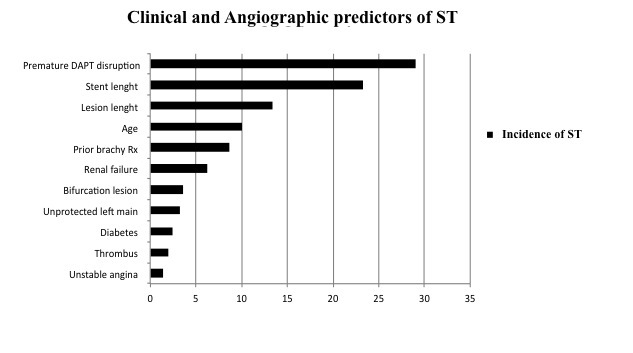
Incidence of stent thrombosis (%) related to clinical and angiographic factors (Adapted from Iakovu I. et al. *JAMA. 2005 May 4;293(17):2126–30.*)

In PCI patients, there is a higher thrombotic risk, and the majority of thrombotic complications occur when metal stent strut and/or polymeric surface are not completely healed. Noteworthy, this high risk of thrombus formation is particularly harmful in a surgical setting, considering the prothrombotic and inflammatory blood properties, the increased levels of cytokines, neuroendocrine inflammatory mediators, platelet counts/adhesiveness, and the impaired fibrinolysis in patients requiring surgery (Tab. 1) (22–24). In particular, ST represents the main potentially catastrophic event that most commonly occurs within the first month after stent implantation. The causes of ST have been carefully investigated, and it is usually related to some specific clinical and angiographic features as reported in Fig. 1 (25–29). A new standard definition of ST was recently proposed by the Academic Research Consortium (ARC), in order to compare the true rates of ST across different trials and registries (Tab. 2) (30). However, in addition to the above-mentioned independent risk factors of thrombotic events, some evidence demonstrate that the premature discontinuation of DAPT represents the main risk factor for ST after BMS (31) or DES (11), and the rebound platelet reactivity after discontinuation of antithrombotic drugs, has been advocated to induce the increased thrombotic risk in PCI patients undergoing surgery (32, 33). Indeed, surgery represents the second cause of early DAPT discontinuation within 1 year (21%) (15). There are several studies updating the ischemic perioperative complications in patients undergoing early surgery after PCI with BMS (16, 34–37) or DES (10, 18, 38–41). Table. 1: varariables associated to pro-thrombotic state in PCI patients undergoing non-cardiac surgery.

The emphasis on DAPT cessation as the trigger for early ischemic events in PCI patients has been recently challenged. A large retrospective study analyzed 41,989 surgical interventions occurring within 24 months after stent implantation (42). Furthermore, the Patterns of non-adherence to Antiplatelet Regimens In Stented patients” (PARIS) registry, analysed whether, the clinical circumstances and reason for discontinuation of DAPT, may have an impact on the cardiovascular risk that follows PCI (43). Interestingly, this registry explored carefully the underlying context in which antiplatelet treatment was discontinued, and patients were stratified into different categories, according to physician-recommended DAPT discontinuation, surgery related brief DAPT interruption, and early DAPT disruption due to uncompliance or bleedings. Surprisingly, physician-guided discontinuation, the most common cause of DAPT withdrawal (40.8% within 2 years), was associated with a significant lower risk of major adverse cardiac events (MACE), probably due to treatment bias in very low thrombotic risk patients. Moreover, brief interruption, occurring in 10.5% of patients for a mean of 6 days, was not associated with an increased rate of thrombotic events, while early disruption, occurring in 14.4% of patients, was associated with a substantial increased risk of MACE, particularly within the first month after PCI. Moreover, the registry reported that, the majority of adverse events occurred while patients were on DAPT, while DAPT disruption did not influenced the overall cardiac outcome. Accordingly, a previous study by Reddy at al. (35) showed that discontinuation of antiplatelet therapy was not associated with perioperative MACE in 56 patients with prior bare-metal stenting.

On the whole, these findings are consistent with other studies suggesting that early DAPT interruption before surgery is not absolutely harmful, if aspirin is continued throughout the surgical intervention, and thienopyridines are resumed as soon as possible after surgery (32, 44). However, whether this concept can automatically be translated to all PCI patients undergoing surgery, remains undefined. Indeed, investigation on the impact of DAPT disruption on the perioperative cardiovascular risk, should also consider the general individual risk profile, the type of CAD (ACS or stable CAD) and both, ischemic and bleeding risk, related to different surgical interventions.

## BLEEDING COMPLICATION IN SURGICAL PATIENTS ON ANTIPLATELET THERAPY

Perioperative bleedings may occur as consequence of surgical procedures or as effect of antiplatelet drug treatment during surgery. Up to date, the causes of bleedings are often a matter of debate (45). Otherwise, there are some surgical procedures that do not increase the risk of bleeding, but that can be associated with bleedings if antithrombotic medications are in use before surgery. The American College of Chest Physicians guidelines for the perioperative management of antithrombotic therapy, identified a group of surgical interventions associated with high bleeding risk in the setting of perioperative anticoagulant and antithrombotic therapy (46), including vascular, visceral and transbronchial surgery. Nevertheless, minor surgical interventions like dental procedures, cataract surgery, dermatologic interventions, as well as angiographic diagnostic procedures or diagnostic endoscopies, can be performed under full antiplatelet therapy, provided that no additional bleeding risk exists (47–49). A meta-analysis collecting 49,590 patients undergoing non-cardiac surgery, estimated that the incidence of bleeding complications, in patients on aspirin therapy, varies from 0% (e.g., in case of skin lesion excision or cataract surgery) up to 75% (e.g., in case of transrectal biopsy) (50). Indeed, aspirin does not increase the severity of bleeding, while influences the number of bleeding events, with exception for intracranial surgery and transurethral prostatectomy. On the other hand, further studies conducted in patients undergoing non-cardiac surgery, reported the increased rate of bleedings with perioperative continuation of both aspirin and P2Y_12_ inhibitors (35, 36, 49). Therefore, it has been suggested the continuation of aspirin or thienopiridine alone, for secondary prevention in many surgical interventions (51). Nevertheless, in patients undergoing surgical procedures in closed anatomical space (e.g. intracranial neurosurgery, posterior chamber of the eye, medullary canal etc.) or if major bleedings are expected, it would rather be necessary to interrupt any kind of antiplatelet agent from 5 to 7 days before surgery (51). Anyway, also the best suitable choice of P2Y_12_ inhibitors plays a role in the management of bleedings. New oral P2Y_12_ inhibitor agents, prasugrel and ticagrelor, are correlated to major bleedings if compared to clopidogrel. Prasugrel, similar to clopidogrel, binds selectively and irreversibly to platelet P2Y_12_ receptor, but has a much rapid and consistent platelet inhibition (52). However, the perioperative use of prasugrel is limited, because of the increased bleeding risk related to its irreversible platelets inhibition. Otherwise, this drug could be used after surgery considering its rapid and potent onset of platelets inhibition. Moreover, in the TRITON-TIMI-38 trial (53), patients with acute coronary syndromes (ACS), referred to coronary artery by-pass grafting (CABG) and randomized to receive prasugrel or clopidogrel, showed a reduction of ischemic events and a rate of TIMI major bleeding greater with prasugrel therapy. Ticagrelor, instead, is a nonthienopyridine agent that directly and reversibly inhibits P2Y_12_ platlet receptor, with better results on platelet aggregation than clopidogrel. The PLATO study (54), a trial comparing ticagrelor versus clopidogrel in ACS patients referred to invasive or non-invasive intervention, demonstrated MACE and ST rates reduction, and a similar rate of TIMI major bleeding to clopidogrel, in patients undergoing CABG within 7 days after last drug intake.

Therefore, the advantage of ticagrelor in perioperative setting could be due to the short half life and to its reversibility (52). Current guidelines (55), thus recommend continuing asprin during surgical procedures, while interrupting clopidogrel or ticagrelor 5 days before surgery, and prasugrel 7 days before surgery. However, in case of cardiac surgery, it is essential to consider the additional bleeding risk factors like heparinization, pump-induced platelet dysfunction and impaired fibrinolysis (56), which further influence the antithrombotic drugs management.

## BRIDGING THERAPY

The perioperative antiplatelet therapy management usually requires the interruption of both aspirin and ADP receptor inhibitor. However, if DAPT is strictly required until surgical intervention, short-acting platelet antagonists should be considered, up to few hours before surgery, as “bridging” therapy. The rationale of the bridging therapy is to reduce the risk of perioperative bleedings, promising ischemic prevention both before surgery and in the early postoperative period, when many surgeons avoid to re-introduce DAPT immediately because of the high bleeding risk. Unfractioned heparins or low molecular weight heparins (LMWH) are not optimal bridging agents, because endovascular thrombosis depends on platelets activation rather than on coagulation cascade (57). Otherwise, glycoprotein IIb/IIIa (GpIIb/IIIa) inhibitors, such as the synthetic peptides eptifibatide (58) or tirofiban (59), hold some characteristics of an ideal bridging agent, including rapid activity, potent antiplatelet effects and short effect duration, except abciximab, that causes a prolonged irreversible platelets aggregation inhibition, and thus should not be used perioperatively (60). However, there are few discordant studies about their efficacy as bridging drugs, and the role of GpIIb/IIIa inhibitors requires more investigations. In addition, the BRIDGE trial (61) recently considered the cangrelor, a reversible P2Y_12_ receptor inhibitor, as an ideal bridging agent in patients undergoing coronary artery by-pass graft (CABG), showing that it is valuable in this specific clinical setting because of its intravenously administration, with rapid onset and offset. Whether these findings hold good also for other kind of non-cardiac surgical interventions, is still a hypothesis, but not a proof yet.

## DEDICATED DEVICES

The introduction of second generation DES in the clinical practice, demonstrated a favourable safety profile in terms of low rate of ST and shorter DAPT duration, when compared to first generation DESs. Up to date, DESs are being used in high-risk lesions, even if several reports have suggested that they may be associated with delayed endothelialization, localized hypersensitivity reactions, and late ST (62–64). In the Efficacy of Xience/Promus Versus Cypher to Reduce Late Loss After Stenting (EXCELLENT) trial (65), 6-month DAPT did not increase the risk of target vessel failure at 12 months after DES, when compared to 2-month DAPT in stratified analyses of patients receiving everolimus- or sirolimuseluting stents. In the Real Safety and Efficacy of 3 month dual-antiplatelet Therapy following Endeavor zotarolimus-eluting stent implantation (RESET) trial (66), a strategy of 3 month-DAPT following Endeavor zotarolimus-eluting stent, was found to be non-inferior in comparison with 12 month-DAPT following other DES, for the primary composite endpoint of cardiovascular death, MI, ST, target vessel revascularization (TVR), or bleeding at 12 months. Lastly, second-generation Xience-Prime and Xience-V everolimus-eluting stents, have recently gained the European CE Mark approval for the use with DAPT for at least 3 months, and the Resolute Integrity zotarolimus-eluting stent for 1 month, which represent the shortest DAPT duration for any major DES in Europe. Nowadays, several new devices that could overcome many limitations of BMS and DES, reducing the risk of ST and requiring shorter DAPT duration, have been developed, including DES with biodegradable polymer which combine the advantage of the early antistenotic benefit of a standard DES and the long-term safety benefit of a BMS. Notheworthy, these new devices, thanks to advanced drug delivery systems and different struts, could be more suitable in patients referred to early surgery after PCI. Indeed, the newer polymer free stent CID OPTIMA new-generation DES, combines the patented polymer-free drug reservoir and the proven antithrombotic and prohealing integral carbofilm coating. The OPTIMA has no drug polymer, and the antiproliferative agent used (tacrolimus) (67), is carried directly on the stent outer surface and released inside the wall vessel (68). Two randomized studies investigated clinical outcome of Janus Flex TES implantation with shorter DAT duration, the JUPITER I study (69) and the JUPITER II trial (70). Both trials demonstrated the safety of this device, without abruption of thrombotic events, despite only 2- or 6-months DAPT course, respectively. Furthermore, the Multicentre registry with Antiplatelet TReatment two–sIX months—MATRIX Study (71), a prospective, multicentre registry, investigated safety and efficacy of Janus Flex TES, at 12-months of follow-up in patients undergoing PCI, with a DAPT duration of 2 or 6 months. Results showed that Janus Flex coronary stenting, followed by short DAPT, is safe and feasible, with no difference between 2- and 6-month DAPT groups. Indeed, another safe and effective option in patients referred to PCI, with known or supposed low compliance to DAPT, or candidated to early surgery, is the Genous Bio-engineered-R stent (GRS) (72). The GRS feature is the ability of capturing circulating endothelial-progenitor cells (EPCs) from the blood stream, ensuring an early stent strut endothelialization (73, 74). Interestingly, a pilot study conducted in patients who need coronary revascularization before undeferrable non-cardiac surgery, showed that, after an average antiplatelet therapy time of 15 days, there were no cardiac events both in the perioperative period and at 30-day follow-up (75). Accordingly, the Antiplatelet therapy after Genous EPC-capturing coroNary stenT implantatiOn — ARGENTO Study (76) investigated the safety and efficacy of GRS with ≤15-day or >15-day DAPT in patients undergoing PCI, with known or expected low compliance to long-term DAPT. No significant differences were reported between different DAPT timing groups, in terms of efficacy and safety end-points (the composit of cardiac death, any myocardial infarction, target vessel revascularization and stent thrombosis). On the whole, these new devices could represent an optional strategy for coronary revascularization in patients who need early surgical intervention because of their shorter DAPT required, but further dedicated studies need to confirm this hypothesis.

## CONCLUSIONS

**Figure 2:**
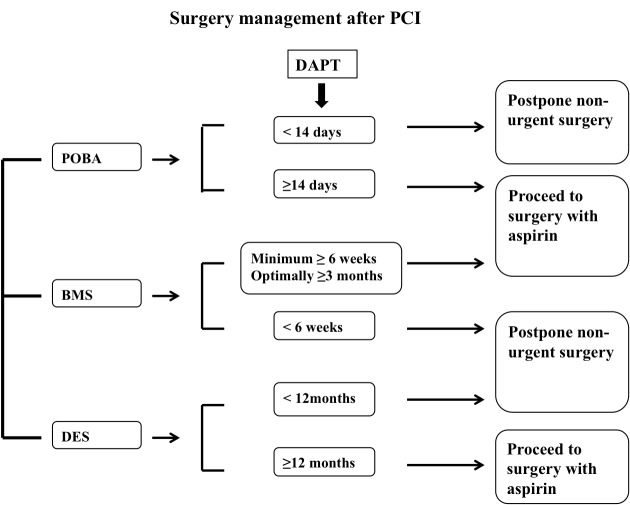
Previous coronary revascularization and surgery management.

Perioperative management of antiplatelet drugs in PCI patients is still a critical topic. However, in case of elective surgery, DES should be avoided, unless a surgery delays up to 12 months following DES deployment. Otherwise, CABG, BMS or POBA should be considered (Fig. 2). In patients with semi-urgent surgery, the decision to early interrupt one, or both, antiplatelet drugs (at least 5–7 days pre-surgery), should undergo multidisciplinary consultation, with the evaluation of the thrombotic and bleeding risk, according to the type of surgical intervention (low or high bleeding risk surgery). Nevertheless, emergent surgery should be performed with full antiplatelet therapy, despite the increased bleeding risk. Indeed, clinical practice guidelines support preventive strategies in order to reduce the risk of DAPT withdrawal in patients referred to surgery, including BMS implantation whenever possible. However, there are new optional strategies to manage PCI patients referred to early surgery, such as the use of dedicated devices, with early healing and brief DAPT, or the perioperative administration of GpIIb/IIIa inhibitors, which represent a potential pharmacological strategy to avoid stent thrombosis and its dramatic effects, without increasing surgical bleedings. Up to date guidelines supply only few patient- and procedure-specific recommendations, in order to help physicians tailoring dedicated treatment strategies, in the setting of early surgery following PCI. Therefore, it is strictly required to consider type of surgical intervention and to balance safety and efficacy profiles of antiplatelets drugs, in this particular scenario.

## Figures and Tables

**Table 1.** Pro-thrombotic features in surgical setting

-	ACUTE CORONARY SYNDROME
-	RENAL DYSFUNCTION
-	DIABETES
-	ADVANCED AGE
-	HYPERCOAGULABLE STATES (E.G. ANEMIA, MALIGNANCY, SURGERY)
-	LOW RESPONSE TO THIENOPIRIDINE
-	HYPERSENSITIVITY TO POLYMER
-	PERSISTENT HIGH PLATELET REACTIVITY
-	STENT UNDEREXPANSION
-	STENT OVERLAPPING
-	RESIDUAL DISSECTION AT THE STENT EDGE
-	TYPE C LESION
-	PLAQUE RUPTURE
-	DELAYED STENT STRUT HEALIN
-	HIGH LEVELS OF FIBRINOGEN AND COAGULATION PROTEINS

**Table 2.** ARC stent thrombosis classification

ARC STENT THROMBOSIS CLASSIFICATION	
**Definite**: symptoms suggestive of ACS and angiographic confirmation of ST. **Probable**: any unexplained death within 30 days or target vessel MI, without angiographic confirmation of ST. **Possible**: any unexplained death after 30 days.	**Early**: 0–30 days post stent implantation: - **Acute**: <24 hours - **Subacute**: 1–30 days **Late**: >30 days **Very late**: >12 months

ARC: Academic Research Consortium; ACS: Acute Coronary Syndrome; ST: Stent Thrombosis; MI: Myocardial Infarction.
